# Use of Head and Chest Circumference Ratio as an Index of Fetal Growth Retardation in Preterm Infants

**DOI:** 10.3390/nu14224942

**Published:** 2022-11-21

**Authors:** Hiromichi Shoji, Yayoi Murano, Yukika Saitoh, Naho Ikeda, Natsuki Ohkawa, Naoto Nishizaki, Ken Hisata, Masato Kantake, Kaoru Obinata, Daisuke Yoneoka, Toshiaki Shimizu

**Affiliations:** 1 Department of Pediatrics, Juntendo University Faculty of Medicine, 2-1-1 Hongo, Bunkyo-ku, Tokyo 113-8421, Japan; 2 Department of Neonatology, Juntendo University Shizuoka Hospital, 1129 Nagaoka, Izunokuni-shi, Shizuoka 410-2295, Japan; 3 Department of Pediatrics, Juntendo University Urayasu Hospital, 2-1-1 Tomioka, Urayasu-shi, Chiba 279-0021, Japan; 4 Department of Neonatology, Juntendo University Nerima Hospital, 3-1-10 Koyadai, Nerima-ku, Tokyo 177-8521, Japan; 5 Division of Biostatistics and Bioinformatics, Graduate School of Public Health St. Luke’s International University, 10-1 Akashi-cho, Chuo-ku, Tokyo 104-0044, Japan

**Keywords:** head circumference to chest circumference ratio, very low birth weight infants, fetal growth restriction, body mass index

## Abstract

We evaluated the relationship between fetal growth in preterm babies using the head circumference (HC)/chest circumference (CC) ratio and other anthropometric parameters at birth and at school age. Data were collected from 187 very low birth weight (VLBW) children born at less than 30 weeks of gestational age (GA) at birth and at 6 years. We assessed the correlation between the HC/CC ratio and body weight (BW), body length (BL), and HC z-scores at birth, and BW, body height (BH), and body mass index (BMI) z-scores at 6 years. Multiple regression analysis showed that BW z-score, BL z-score, and HC z-score at birth were significantly associated with HC/CC at birth. The BMI z-score at 6 years was also significantly associated with HC/CC at birth. The HC/CC ratio at birth is a reliable parameter for evaluating fetal growth restriction and a possible predictor of physical growth in VLBW children.

## 1. Introduction

The prognosis of preterm infants has improved dramatically in recent decades. The mortality rate of extremely preterm infants in Japan is shallow compared to that in other developed countries [1,2]. However, children born extremely preterm are at high risk of adverse long-term clinical and developmental outcomes [3]. Some studies also reported that preterm children have different body shapes than children born at-term [4,5]. In contrast, fetal growth restriction (FGR) describes a fetus that does not grow to its expected biological potential in utero and is the primary cause of infants being small for gestational age (SGA) [6,7]. Growth restriction in utero affects later growth and body composition [8,9]. Children born prematurely are more likely to have a growth restriction than children born at-term [10]. We previously demonstrated that the youngest gestational age (<25 weeks’ gestation) had a greater influence in males on the z-score of anthropometric parameters up to 6 years of age compared to those of older gestational age (25–29 weeks’ gestation) [11].

Anthropometric assessment is essential for monitoring and evaluating the nutritional status and growth of fetuses, newborns, and infants. Fetal growth during pregnancy is indicated by anthropometric criteria at birth, particularly weight. Circumferential dimensions using a tape measure can evaluate nutritional status. For example, mid-upper arm circumference is often measured in children or infants but is rarely assessed at birth. Chest circumference (CC) and head circumference (HC) are simple, reliable, and logistically feasible tools. CC correlates highest with body weight (BW) at birth [12,13,14]. In addition, the HC/CC ratio may also be a better indicator of macrosomia due to maternal diabetes than birth weight [15].

In contrast, cohort studies examining the correlation between SGA and anthropometry among preterm-born children, especially using the HC/CC ratio, are limited. This study aimed to evaluate the relationship between the HC/CC ratio and other anthropometric parameters at birth and 6 years-corrected age (CA) in very-low-birth-weight (VLBW) infants born at less than 30 completed weeks of gestation.

## 2. Materials and Methods

### 2.1. Subjects

This was a multicenter, retrospective study enrolling neonates admitted to neonatal intensive care units (NICUs) in the three affiliated hospitals of Juntendo University (Juntendo University Hospital in Tokyo, Juntendo University Urayasu Hospital in Chiba, and Juntendo University Shizuoka Hospital in Shizuoka) from January 2007 to June 2012. The inclusion criteria of participants were birth weight <1500 g and gestational age (GA) < 30 weeks. GA was determined based on the mother’s last menstrual period and first-trimester ultrasound. According to our nutritional protocol, feeding was typically initiated within the first 8 h after birth (20 mL/kg divided into over 8 feedings per day), and own mother’s breast milk (BM) was the preferred type of feeding. However, when BM was unavailable due to the mother’s unwillingness or inability to provide BM, the infants received preterm formula. Milk intake was increased by about 20 mL/kg daily to 120 mL/kg, at which time the human milk fortifier (HMS-1; nutrient composition/1 g: calories, 3.37 kcal; protein, 0.26 g; Morinaga Milk Industry (Tokyo, Japan)) was added to the BM. Typically, we change from preterm formula to general infant formula when the weight of infants exceeds 2000 g.

We collected the data of BW, body length (BL), HC, CC at birth, BW, and body height (BH) at 6 years of CA (representing the age of the children from the expected date of delivery) from medical records. The ponderal index (PI) (g/cm^3^) at birth or body mass index (BMI) (kg/m^2^) at 6 years were calculated. Sex- and GA-independent z-scores and percentiles for anthropometric parameters at birth (including BL, BW, and CC) were calculated according to the Japanese standard curve, estimated using 2003–2005 data from the Japan Society of Obstetrics and Gynecology registry database [16]. SGA was defined as a z-score for birth weight <10 percentile. The exclusion criteria were infants with congenital diseases, chromosomal abnormalities, and severe cardiac, renal, or endocrine diseases. Infants whose birth weight z-scores were >2 and children who received growth hormone therapy were also excluded from the study. Z-scores for BW, BH, and BMI at 6 years of age were also calculated according to the standard growth chart for children from a national survey conducted in 2000 [17]. An Excel-based program has been developed for plotting both standard curves by the Japanese Society for Pediatric Endocrinology; this software is available on their website [18].

### 2.2. Statistical Analysis

First, we calculated the mean ± standard deviation (SD) to assess patients’ characteristics. Then, to determine the statistical correlation between HC/CC ratio and GA, BW z-score, BL z-score, HC z-score, and PI at birth and BW z-score, BH z-score, and BMI z-score at 6 years of CA, we used Spearman’s rank correlation coefficient analysis. Furthermore, we examined whether HC/CC, GA, and sex are associated with anthropometric measures at birth and 6 years of CA using regression analysis. First, using a simple univariate regression model, we examined the association between HC/CC, GA, and sex and anthropometric measures, including BW z-score, BL z-score, HC z-score, and PI at birth and BW, BH, BMI z-score at 6 years of CA. Further, we performed a multiple regression analysis using the covariates that showed significant associations in the simple univariate regression analysis. Statistical significance was set at *p* < 0.05. Finally, to evaluate the predictive performance of the HC/CC ratio for SGA, the receiver operating characteristic (ROC) curve and the area under the curve (AUC) were calculated. The best predictive value (and the associated threshold value) was calculated using the Youden index. All statistical analyses were performed using Stata version 15.1 (StataCorp, College Station, TX, USA).

## 3. Results

During the study period, 529 VLBW infants with GA < 30 weeks were admitted to the NICU of three hospitals, and 502 preterm infants met the inclusion criteria at birth. Of these, 412 patients were discharged from the NICU. We collected data from 197 children up to 6 years of CA and excluded 10 children who received growth hormone therapy. We analyzed the 187 remaining children, of whom 100 were males and 87 were females. Specific complications were as follows: SGA (19.8% of subjects), intraventricular hemorrhage (3.2%), necrotizing enterocolitis (2.1%), chronic lung disease (68.4%), and home oxygen therapy (18.2%). Table 1 represents the anthropometric parameters at birth and 6 years of CA of subjects in this study. The mean gestational age was 26.8 (22.4–29.9) weeks, and the mean birth weight was 843 (328–1462) g. The mean body weight was 17.5 (12.7–26.2) kg, and the mean body height was 109 (97–122) cm at 6 years of CA.

**Figure 1:**
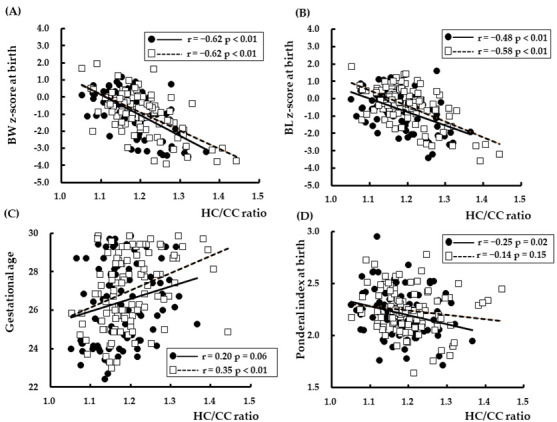
Relationships between HC/CC at birth and (**A**) BW z-score, (**B**) BL z-score, (**C**) HC z-score, and (**D**) ponderal index at birth. BW: body weight, BL: body length, HC: head circumference, CC: chest circumference. The r values show Spearman’s rank correlation coefficients. ●: Girls, □: Boys.

**Figure 2:**
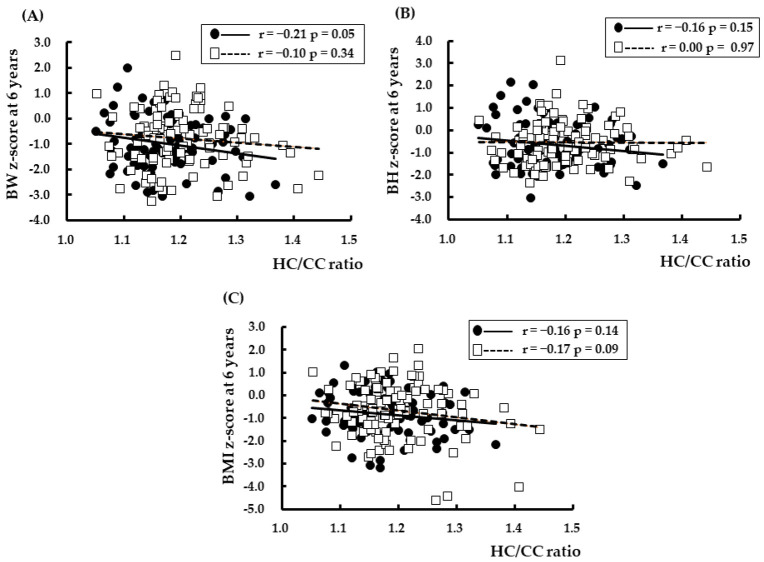
Relationships between HC/CC at birth and (**A**) BW z-score, (**B**) BH z-score, and (**C**) BMI z-score at 6 years corrected age. BW: body weight, BL: body length, HC: head circumference, BMI: body mass index. The r values show Spearman’s rank correlation coefficients. ●: Girls, □: Boys.

Spearman’s rank correlation coefficient analysis showed that the HC/CC ratio was significantly correlated with BW z-score, BL z-score, HC z-score, PI at birth, BMI z-score at 6 years, and GA (Figure 1 and Figure 2). The relationships between BW z-score, BL z-score, HC z-score, PI at birth, and HC/CC ratio at birth are shown in Figure 1. The relationships between BW z-score, BH z-score, and BMI z-score at 6 years of CA and HC/CC ratio at birth are shown in Figure 2.

The simple univariate regression analysis showed that the statistical association between BW z-score, BL z-score, HC z-score, PI, and HC/CC ratio was observed at birth. The associations between BW z-score at birth and GA, HC z-score at birth and sex, and PI at birth and GA were also seen (Table 2). Although there was no significant association between BW and BH z-scores at 6 years of CA and the HC/CC ratio at birth, the BMI z-score at 6 years of CA was significantly associated with HC/CC ratio at birth (Table 2).

The results of the following multiple regression analysis using covariates that showed statistical significance in the simple regression analysis are presented in Table 3. BW z-score and PI were associated with HC/CC ratio. HC-z-score was associated with both HC/CC ratio and sex. The BMI z-score at 6 years of CA was also significantly associated with the HC/CC ratio at birth and GA.

**Figure 3:**
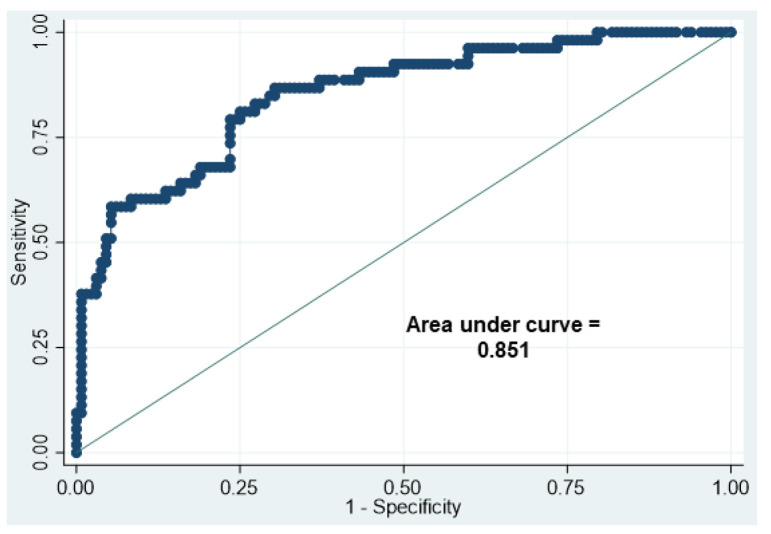
Receiver operator characteristic curve of head circumference/chest circumference ratio for the prediction of being small for gestational age.

The AUC of the HC/CC ratio for the prediction of SGA was 0.851. The HC/CC ratio showed a sensitivity of 86.8% and a specificity of 68.9% at the cut-off value of 1.18 (Figure 3).

## 4. Discussion

To the best of our knowledge, this is the first study to evaluate the relationship between the HC/CC ratio and other anthropometric parameters at birth and at 6 years of CA among VLBW children. We demonstrated that the HC/CC ratio was strongly influenced by SGA and was associated with physique at 6 years.

The most common fetal biometric parameters were biparietal diameter, HC, abdominal circumference, and femur diaphysis length, as measured by ultrasound. These biometric measurements could help estimate fetal weight using various formulas to evaluate FGR [19]. At birth, BW, BL, and HC z-scores, rather than specific anthropometric measurements, are usually assessed to evaluate SGA among preterm infants, to exclude the possibility of GA bias. On the other hand, SGA is commonly defined as BW at birth below the tenth percentile.

Circumferential dimensions are noninvasive, reproducible, and low-cost methods that can be performed quickly. HC is strongly correlated with length at birth, as well as later in infancy and early childhood [20], and with cognition throughout early school age [21,22]. On the other hand, CC is rarely measured in early childhood. The use of CC as a surrogate for identifying low-birth-weight (LBW) infants has been recommended in several studies, mainly due to its high sensitivity in diagnosing LBW. This is due to the simplicity of the procedure, as the nipple line is an obvious landmark for measurement, making measurements less prone to interobserver or intraobserver variability [23]. A meta-analysis performed in Japan showed that CC is a good indicator of LBW [24]. CC and HC were used to identify LBW preterm neonates in Ethiopia, and the cut-off points with the best sensitivity and specificity were identified as 30 cm and 31 cm for CC and HC, respectively [25]. Gidi et al. recently conducted a similar study in Ethiopia, and the optimal cut-off point indicative of LBW was ≤31.2 cm for CC [23].

Moreover, the result of the ROC curve in this study showed that the HC/CC ratio of 1.18 is one of the good predictors for SGA in VLBW infants. The rationale behind using the HC/CC ratio is as follows: HC is generally larger than CC at birth. After birth, HC does not increase as rapidly as CC does. In well-nourished children, CC becomes larger than HC between 6 and 12 months of age, producing a mean HC/CC ratio of less than 1 [26]. HC and CC are important indicators of growth status in infancy and early childhood since severe undernutrition early in life is associated with marked retardation in the growth of both HC and CC [27]. In this study, the HC/CC ratio at birth was associated with the BMI z-score at 6 years. Our previous study also demonstrated that SGA affects anthropometric parameters for 6 years among the same population assessed by ANOVA [28]. It is necessary to consider whether the same evaluation is possible for Japanese at-term infants. Furthermore, the relationship between changes during early infancy in the HC/CC ratio and the prognosis of the physique should be examined.

This study had some limitations. We did not correct potential variables such as parents’ age, parents’ physique, maternal complications during pregnancy, or nutritional management and feeding during infancy. Furthermore, the sample size was small, and the follow-up rate of participants was low (45%). Most of the correlations in this study were not strong due to the small sample size. We assume that study subjects with a good prognosis stopped coming to the hospital for follow-up checkups. We believe that this may not have influenced our results.

## 5. Conclusions

Our study suggests that the HC/CC ratio is a reliable parameter for evaluating SGA and a possible predictor of physical growth in VLBW children born at <30 weeks of GA. Further research is needed to demonstrate the correlation between the HC/CC ratio and growth and long-term complications in preterm infants.

## Figures and Tables

**Table 1:** Anthropometric indices of children at birth and 6 years of corrected age.

		Boys		Girls	
		All (100)	SGA (30)	All (87)	SGA (23)
At birth	Gestational age (weeks)	27.0 ± 1.9 (23.0–29.8)	27.5 ± 1.7 (24.9–29.9)	26.5 ± 2.1 (22.4–29.8)	27.2 ± 1.6 (23.6–29.9)
	Body weight (g)	888 ± 260 (338–1462)	718 ± 190 (338–1104)	791 ± 261 (328–1426)	636 ± 191 (328–1072)
	Body weight z-scores	−0.9 ± 1.2 (−3.9–1.9)	−2.4 ± 0.8 (−3.9–−1.4)	−0.8 ± 1.3 (−3.4–1.2)	−2.7 ± 0.7 (−3.5–−1.3)
	Body length (cm)	33.8 ± 3.4 (24.0–40.0)	32.0 ± 3.1 (24.0–40.0)	32.6 ± 3.7 (25–40.5)	30.7 ± 3.3 (25–37.8)
	Body length z-scores	−0.4 ± 1.1 (−3.6–1.8)	−1.5 ± 1.0 (−3.6–0.4)	−0.6 ± 1.1 (−3.4–1.6)	−2.0 ± 0.7 (−3.4–−0.2)
	Head circumference (cm)	24.4 ± 2.4 (19.6–29)	23.5 ± 2.0 (20–27.9)	23.2 ± 2.4 (18.5–30.2)	22.6 ± 2.1 (18.5–27.0)
	Head circumference z-scores	0.0 ± 0.8 (−3.6–1.8)	−0.8 ± 0.7 (−2.2–0.4)	−0.4 ± 0.8 (−2.3–2.3)	−1.2 ± 0.5 (−2.3–−0.1)
	Chest circumference (cm)	20.4 ± 2.2 (14.0–25.0)	18.7 ± 1.9 (14.0–23.3)	19.7 ± 2.3 (15–26.5)	18.3 ± 2.0 (15–22.5)
	HC/CC ratio	1.2 ± 0.7 (1.1–1.4)	1.3 ± 0.1 (1.1–1.4)	1.2 ± 0.1 (1.1–1.4)	1.2 ± 0.1 (1.1–1.4)
	Ponderal index (g/cm^3^)	2.2 ± 0.2 (1.6–2.8)	2.2 ± 0.2 (1.6–2.8)	2.2 ± 0.2 (1.7–3.0)	2.1 ± 0.3 (1.7–2.7)
At 6 years	Body weight (g)	17.8 ± 2.8 (12.9–26)	16.9 ± 2.7 (12.9–24)	17.0 ± 2.3 (12.7–26.2)	16.4 ± 2.0 (12.7–19.4)
	Body weight z-scores	−0.8 ± 1.2 (−3.2–2.5)	−1.3 ± 1.1 (−3.1–1.2)	−1.0 ± 1.0 (−3.0–2.0)	−1.3 ± 1.0 (−3.0–0.1)
	Body height (cm)	109 ± 4.8 (100–122)	109 ± 4.9 (100–120)	109 ± 4.9 (97.4–121)	107 ± 4.1 (100–115)
	Body height z-scores	−0.5 ± 1.0 (−2.4–3.1)	−0.8 ± 0.8 (−2.3–0.9)	−0.7 ± 1.0 (−3.0–2.2)	−0.9 ± 0.9 (−2.5–1.0)
	BMI (kg/m^2^)	14.7 ± 1.5 (11.4–19.6)	14.3 ± 1.7 (11.4–19.6)	14.3 ± 1.2 (11.9–17.9)	14.1 ± 1.1 (12.5–16.5)
	BMI z-scores	−0.7 ± 1.2 (−4.6–2.1)	−1.2 ± 1.5 (−4.6–2.1)	−0.8 ± 1.0 (−3.1–1.3)	−1.0 ± 0.9 (−2.4–0.6)

Data are presented as means ± standard deviation and range. HC: head circumference, CC: chest circumference, BMI: body mass index, SGA: small for gestational age.

**Table 2:** Single regression analysis on anthropometric indices at birth and 6 years corrected age.

	Variables	Coefficient	95% CI	*p*
**At birth**				
Body weight z-score	HC/CC ratio	−11.08	−13.13–−9.03	<0.01
	Gestational age	−0.13	−0.22–−0.04	<0.01
	Sex	0.13	−0.22–0.04	0.73
Body length z-score	HC/CC ratio	−8.12	−10.06–−6.19	<0.01
	Gestational age	−0.07	–0.15–0.01	0.08
	Sex	−0.19	−0.51–0.13	0.23
HC z-score	HC/CC ratio	−2.28	–3.99–−0.57	<0.01
	Gestational age	0.04	−0.02–0.10	0.19
	Sex	−0.41	−0.65–−0.17	<0.01
Ponderal index	HC/CC ratio	−5.73	−10.15–−1.32	0.01
	Gestational age	−0.16	−0.32–−0.01	0.04
	Sex	−0.25	−0.88–0.38	0.43
At 6 years				
Body weight	HC/CC ratio	–2.01	−4.26–0.24	0.08
	Gestational age	0.12	0.04–0.19	<0.01
	Sex	−0.21	−0.53–0.10	0.19
Body height	HC/CC ratio	−0.92	−2.97–1.13	0.38
	Gestational age	0.07	0.00–0.14	0.05
	Sex	−0.13	−0.41–0.16	0.39
BMI z-score	HC/CC ratio	−2.43	−4.68–−0.18	0.03
	Gestational age	0.09	0.01–0.17	0.02
	Sex	−0.15	−0.47–0.17	0.37

HC: head circumference, CC: chest circumference, BMI: body mass index.

**Table 3:** Multiple regression analysis on anthropometric indices at birth and 6 years corrected age.

	Variables	Coefficient	95% CI	*p*
At birth				
Body weight z-score	HC/CC ratio	−10.91	−13.06–−8.76	<0.01
	Gestational age	−0.02	−0.10–0.05	0.59
HC z-score	HC/CC ratio	−2.75	−4.41–−1.08	<0.01
	Sex	−0.47	−0.70–−0.23	<0.01
Ponderal index	HC/CC ratio	−4.76	−9.36–−0.15	0.04
	Gestational age	−0.12	−0.28–0.04	0.15
At 6 years				
BMI z-score	HC/CC ratio	−3.45	−5.75–−1.15	<0.01
	Gestational age	0.12	0.04–0.20	<0.01

HC: head circumference, CC: chest circumference, BMI: body mass index.

## Data Availability

Not applicable.
